# Validation of a Wearable Virtual Reality Perimeter for Glaucoma Staging, The NOVA Trial: Novel Virtual Reality Field Assessment

**DOI:** 10.1167/tvst.13.3.10

**Published:** 2024-03-15

**Authors:** Chris Bradley, Iqbal Ike K. Ahmed, Thomas W. Samuelson, Michael Chaglasian, Howard Barnebey, Nathan Radcliffe, Jason Bacharach

**Affiliations:** 1 Wilmer Eye Institute, Johns Hopkins University School of Medicine, Baltimore, MD, USA; 2 Department of Ophthalmology and Vision Sciences, University of Utah, Salt Lake City, UT, USA; 3 Department of Ophthalmology and Vision Sciences, University of Toronto, Toronto, Ontario, Canada; 4 Minnesota Eye Consultants, Excelsior, MN, USA; 5 Illinois College of Optometry, Chicago, IL, USA; 6 Specialty Eyecare Centre, Bellevue, WA, USA; 7 Department of Ophthalmology, University of Washington, Seattle, WA, USA; 8 New York Eye Surgery Center, Bronx, NY, USA; 9 North Bay Eye Associates, Petaluma, CA, USA

**Keywords:** visual field, glaucoma, virtual reality

## Abstract

**Purpose:**

Compare estimated sensitivities of SITA-Standard to the RATA-Standard algorithm of the Radius virtual reality perimeter (VRP), and measure concordance in glaucoma staging.

**Methods:**

One hundred adult glaucoma patients—half with suspect or mild glaucoma, and half with moderate or severe—from five clinics performed four 24-2 visual field tests during a single visit, two with the Humphrey Field Analyzer (HFA) and two with Radius, in randomized order: HRHR or RHRH. Only one eye was tested per participant. We used the Wilcoxon rank sum test with Bonferroni correction to compare distributions of estimated sensitivities across all 54 test locations over the 15 to 40 dB measurement range of the Radius. Weighted kappa measured concordance in glaucoma staging between two masked glaucoma experts using Medicare definitions of severity.

**Results:**

A total of 62 OD and 38 OS eyes were tested. Estimated sensitivities for SITA-Standard and RATA-Standard were not significantly different for OD, but were for OS—likely because of SITA-Standard OD and OS being significantly different in our sample, but not for RATA-Standard. Low agreement was observed between 15 to 22 dB. Concordance in glaucoma staging was high for both graders: kappa = 0.91 and kappa = 0.93. Average test duration was 298 seconds for RATA-Standard and 341 seconds for SITA-Standard. The correlation in mean deviation was 0.94.

**Conclusions:**

Estimated sensitivities of RATA-Standard are comparable to SITA-Standard between 23 to 40 dB with high concordance in glaucoma staging.

**Translational Relevance:**

Radius VRP is statistically noninferior to HFA when staging glaucoma using Medicare definitions.

## Introduction

The measurement of visual fields is an integral component of glaucoma diagnosis and long-term care of glaucoma patients. Typically, automated perimeters such as the Humphrey field analyzer (HFA) are used to measure visual fields (VF). Although the HFA is currently considered to be the clinical standard in static automated perimetry, there are drawbacks to its use. Notably, the HFA is a bulky device that requires one-on-one clinical oversight to measure VFs, and patients often dislike VF testing with the HFA.^1^

Recent technological advances have made it possible to perform static automated perimetry without clinician assistance using virtual reality (VR) in a lightweight wearable headset.^2^^–^^6^ Unlike vision tests performed on a tablet or a personal computer,^7^^,^^8^ headset-based virtual reality perimeters (VRP) enable precise control of key visual field test parameters such as background luminance (by controlling ambient light levels using a light shield) and the average size of the retinal image produced by the test stimulus (by controlling average viewing distance). Patient satisfaction is also enhanced with wearable VRPs.^6^^,^^9^

Wearable VRPs have the potential to improve the accuracy of diagnosing glaucoma and identify worsening by enabling more frequent VF testing than is practical with conventional perimeters. A recent study demonstrated that at current VF examination frequencies—once every 466 ± 232 days in a sample of mor than 20,000 glaucoma or glaucoma suspect eyes—the accuracy of detecting both moderate and rapid glaucoma worsening over a two-year period is often less than 50%.^10^ The recommendation in the study to vastly increase VF examination frequency is, however, difficult to implement with conventional perimeters that require in-clinic visits, with one technician measuring one VF at a time. Wearable VRPs offer a possible solution: more frequent VF testing can be performed either in clinic or remotely, and clinical workflow can be improved by enabling a single technician to simultaneously administer multiple VFs in an office space that has not been fully dedicated to testing, such as a waiting room or dilating area.

A major barrier to the adoption of wearable VRPs in clinical practice is the lack of peer-reviewed validation studies demonstrating statistical noninferiority to a clinical standard such as the HFA. Statistical noninferiority should be demonstrated for estimated sensitivities at each test location, not just for summary statistics such as mean sensitivity or mean deviation (MD) as is often done in VRP validation studies.^3^^–^^7^ Concordance in summary statistics does not imply concordance in estimated sensitivities at individual test locations, which is crucial in the management of glaucoma. Furthermore, no commonly used statistical test assesses the biological plausibility of observed defects. Therefore it is important to measure concordance in glaucoma staging by expert clinicians in addition to performing hypothesis tests on estimated sensitivities at each individual test location. Thus far, no published VRP validation studies have satisfied both of these criteria.

In this study, we compare estimated sensitivities of SITA-Standard to the RATA-Standard algorithm of the Radius VRP at all 54 test locations of the 24-2 test pattern and measure concordance in glaucoma staging. Unlike many other commercially available wearable VRPs that lower background luminance into the mesopic range (between 0.01 and 3 cd/m^2^), the Radius VRP presents the Goldmann III stimulus on the same 10 cd/m^2^ background luminance as the HFA, making it a good candidate for demonstration of statistical noninferiority.

## Methods

### The Radius VRP

**Figure 1.**
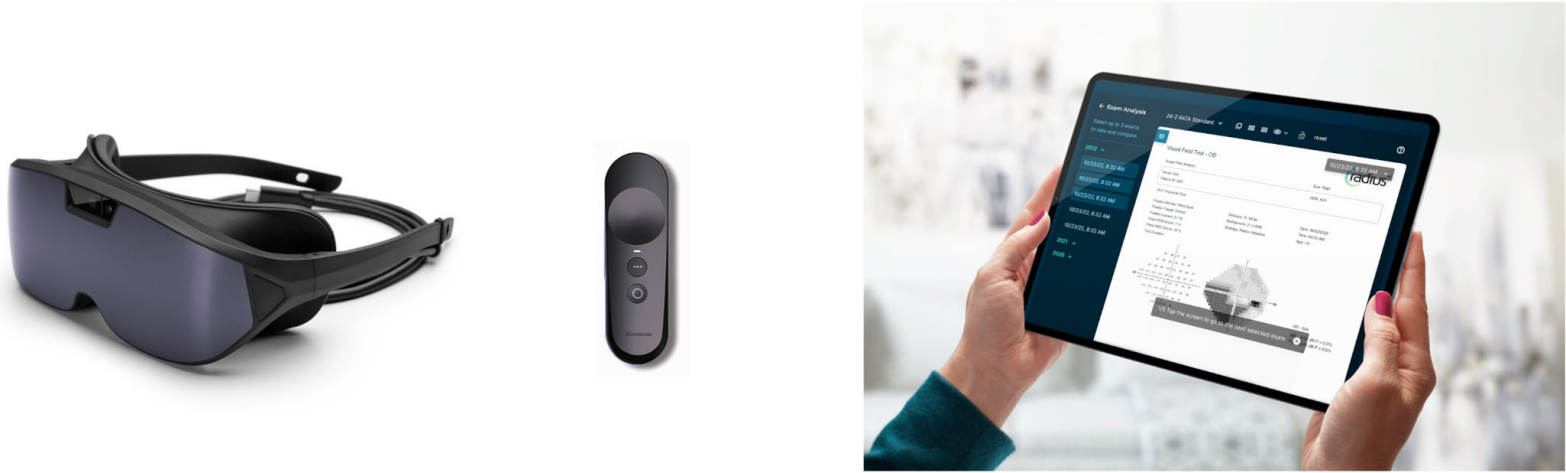
The Radius headset, hand controller and tablet.

The Radius VRP tested in this study (model name: Inspire; model no. ISP0001) consists of a lightweight headset (approximately 170 g) with two 1600 × 1600 liquid crystal displays, a refresh rate of 60 Hz, a 100° field of view, and a maximum brightness of 85 cd/m^2^. A handheld Bluetooth VR controller is used to signal the detection of the Goldmann III stimulus during VF testing. The headset is designed for an interpupillary distance of 61 to 69 mm, and power to the headset is provided by a Samsung tablet, which is used by a technician to monitor testing in real-time (Fig. 1). Because of current power limitations the measurement range of the Radius is limited to 15 to 40 dB on the HFA scale. This means that sensitivities less than 15 dB cannot be discriminated on the Radius and are shown as <15 dB. On the HFA, sensitivities less than 0 dB (the dB scale can go negative) cannot be discriminated and are shown as <0 dB. Similarly, the Radius shows >40 dB for all sensitivities estimated to be greater than 40 dB, whereas the HFA has a 50 dB upper limit.

We note that other commercially available wearable VRPs that lower background luminance into the mesopic range do not have the 15 to 40 dB limitation of the Radius. Lowering background luminance increases the contrast of the stimulus, which increases measurement range. However, lowering background luminance into the mesopic range means that a different component of the human visual system is being tested than the photopic testing done by the HFA. In the mesopic range, both rods and cones are active, whereas in the photopic range (>3 cd/m^2^) only cones are active.

The RATA-Standard algorithm of the Radius is proprietary, but based on well-known methods for estimating contrast sensitivity (visual field tests estimate contrast sensitivity at many test locations). A staircase procedure is used to specify the contrast level of the next presented stimulus at each test location, and a Weibull psychometric function is fit to all subject responses at each test location after each trial. Bayesian inference is used to update sensitivity estimates, starting from a bimodal prior probability distribution of sensitivities—one peak representing normal sensitivities and the other peak representing dense scotomas. RATA-Standard uses the SITA growth pattern, which divides all 54 test locations of the 24-2 test pattern into four groups (e.g., group 1 has four test points, one in each quadrant), with testing group N completed before testing group N + 1, for N ≥ 1. At each of the 54 test locations, testing during the “growth pattern” phase stops if one of four conditions is satisfied: (1) the standard error of the sensitivity estimate is smaller than some predetermined criterion; (2) the subject detects the lowest possible non-zero contrast level twice in a row; (3) the subject fails to detect the highest possible contrast level twice in a row; (4) the maximum number of reversals is reached (three at non-nasal test locations and four at nasal test locations). After the “growth pattern” phase is completed, retesting occurs at every test location where the estimated sensitivity is −7 dB or lower relative to all immediate neighbors. During retesting, the staircase pattern continues for two extra reversals. The final estimated sensitivity at each test location is the brightness level of the stimulus at which the fitted Weibull function predicts a 50% probability of detection.

The Radius also estimates fixation losses (FL), false-positive percentages (FP%) and false-negative percentages (FN%). Unlike some other commercially available VRPs, the Radius does not have eye tracking and uses dedicated FL trials (stimuli periodically presented at the presumed location of the blind spot) to test whether a test subject's fixation is close to the fixation stimulus presented at the center of the display. FP% is calculated as the percentage of trials in which the test subject responded within the inter-trial interval—an interval between trials that lasts 100–200 ms—or during the 200 ms presentation of the Goldmann III stimulus (a response within 180–200 ms of stimulus onset is likely not a response to the stimulus).^11^ FN% is calculated using dedicated FN trials that present a stimulus with an intensity at least 7 dB lower than the estimated sensitivity—such testing occurs strategically at test locations where sensitivities are already estimated. Although certain details of the RATA-Standard algorithm and how the Radius calculates FL, FP%, and FN% are proprietary, the Radius VRP is commercially available, enabling other researchers to replicate this study.

### Participants

We aimed to recruit at least 100 adult glaucoma or glaucoma suspect patients from five different clinics. Recruitment lasted from October 2022 to June 2023. The goal was for half the study participants to be previously diagnosed with moderate or severe glaucoma, and the other half with suspect or mild glaucoma, using Medicare criteria. For all candidate study participants, historic HFA VFs within one year of the recruitment date were examined by the study examiners (C.B., J.B.) to confirm either a normal VF or a classic glaucoma defect. Only study participants whose historic HFA VFs were sufficiently reliable were included—FL, FP and FN percentages were all less than 20%. If historic HFA VFs of both eyes met the inclusion and exclusion criteria, the right eye was chosen as the test eye. The study protocol was approved by the WCG Institutional Review Board and adhered to the Declaration of Helsinki, and all study participants provided informed consent.

To be eligible for the study, study participants were required to have a best-corrected visual acuity of 20/60 (0.477 logMAR) as measured by ETDRS in their test eye; between −6.00 to 5.00 sphere refractive error; astigmatism ≤2.0 diopters; interpupillary distance of 61 to 69 mm; no history of systemic or ocular condition (other than glaucoma or mild to moderate cataract) affecting visual function in the test eye (e.g., strabismus, amblyopia, myasthenia gravis, severe macular pathology, thyroid eye disease, narrow or closed angle glaucoma); no recent cerebrovascular accident; no ocular surgery within six months of enrollment; no hearing impairment affecting the ability to hear auditory features in the headset; no clinical trial participation within the last 90 days that may interfere with study results.

### Test Procedure and Statistical Analysis

Each study participant performed a total of four 24-2 VF tests on one test eye—only one eye was tested per participant—during the same visit: two with the SITA-Standard algorithm of the HFA (H) and two with the RATA-Standard algorithm of the Radius (R), with test order randomized to HRHR or RHRH. Breaks were provided between tests as necessary. To reduce any learning effect for the Radius (all study participants were familiar with the HFA but new to the Radius), study participants performed a short 30 second practice test with the non-test eye on the Radius first. No feedback on test performance was provided for any test. One difference between HFA and Radius testing was that the test eye was corrected for refractive error (both spherical and cylindrical) using the standard trial lenses for the HFA—this was not done for the Radius.

Estimated sensitivities of SITA-Standard and RATA-Standard were compared over a common 15 to 40 dB measurement range. One issue was how to analyze <0 dB on the HFA and <15 dB on the Radius, because these represent ranges of sensitivities instead of a single estimated value. Because most clinicians are likely to interpret <0 dB on the HFA as 0 dB—many may not even be aware that the dB scale can go negative—we set all <15 dB to 15 dB. In addition, all sensitivities >40 dB were set to 40 dB. Although this approach creates an artificial floor and ceiling, it enables hypothesis testing of distributions of estimated sensitivities at each test location without excluding any data. We used the Wilcoxon rank sum test with Bonferroni correction for multiple comparisons (there are 54 test locations) to test whether the distributions of estimated sensitivities at any test location are significantly different between SITA-Standard and RATA-Standard.

The Wilcoxon rank sum test with Bonferroni correction was also used to compare sensitivity distributions of test and retest, as well as OD versus OS for both SITA-Standard and RATA-Standard. Deming regression was used estimate the slope of the best-fitting line between estimated sensitivities across all test locations. We used Deming regression instead of linear regression because it allows for error in both x- and y-axes (both HFA and Radius have measurement errors). Both Deming regression and the Pearson correlation coefficient were used to compare MD values, whereas the Wilcoxon rank sum test (without Bonferroni correction) was used to compare distributions of test duration, FL, FP, and FN. All analysis was done using the R programming language.

### Concordance in Glaucoma Staging

Our second primary endpoint was measuring concordance in glaucoma staging given the estimated sensitivities. Two masked glaucoma experts (H.B. and N.R.) were tasked with staging all test-retest pairs of SITA-Standard and RATA-Standard VFs using Medicare definitions for mild, moderate and severe glaucoma.^12^ Graders staged test-retest pairs of VFs rather than individual VFs to make it easier to identify artifacts—both graders were intimately familiar with the HFA but not with the Radius, which may produce a different type of artifact. Test-retest pairs of VFs were staged in random order, one at a time, with no patient information allowing graders to identify the study participant or match HFA and Radius VFs to the same individual. Graders were allowed to use expert judgment instead of strict Medicare definitions when appropriate (e.g., if a defect looked like an artifact or did not make biological sense) and could examine all test-retest pairs of VFs as many times as desired before submitting their final staging.

**Figure 2.**
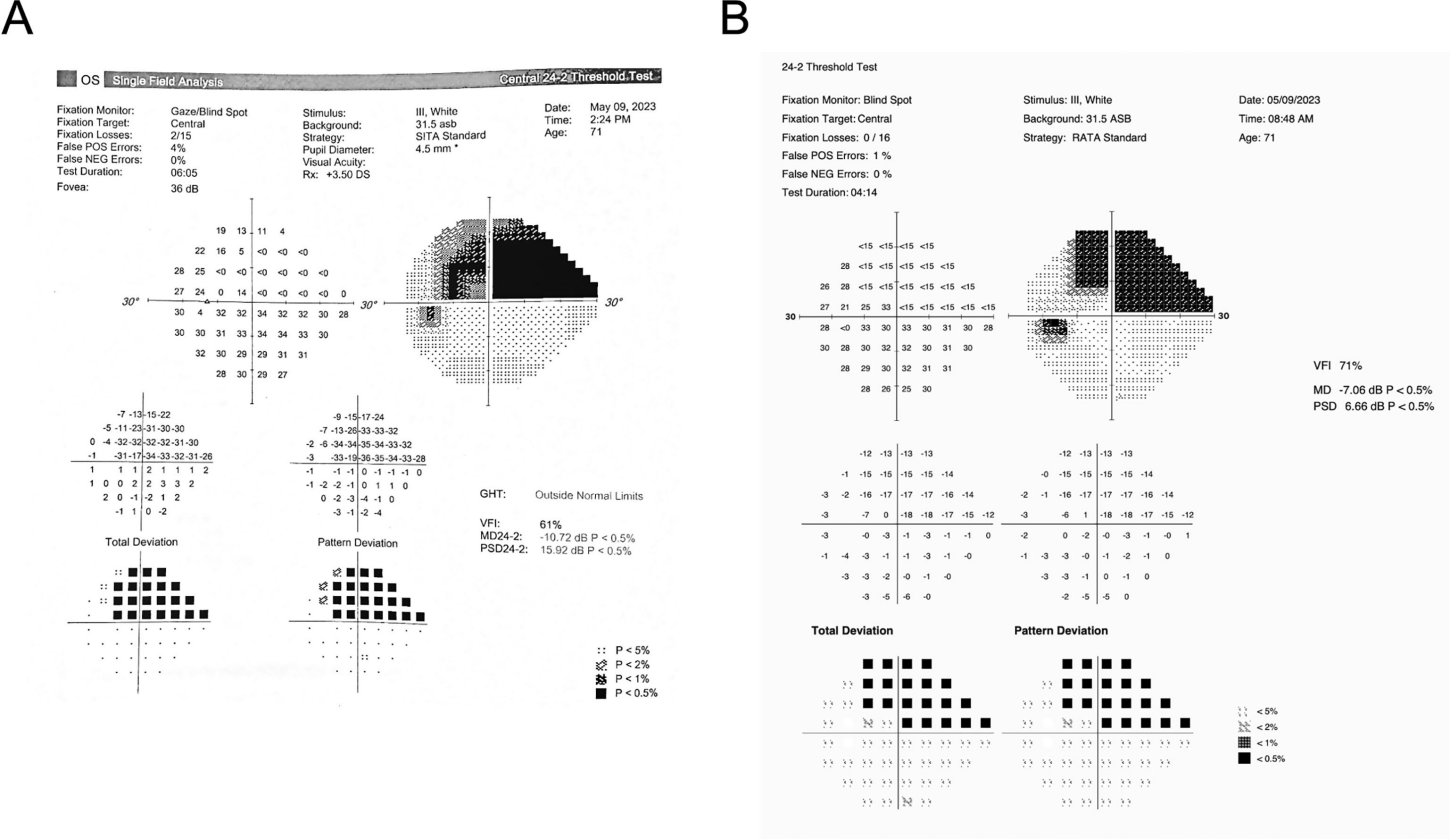
Example outputs for the same eye for the (**A**) HFA using the SITA-Standard algorithm and the (**B**) Radius using the RATA-Standard algorithm.

The Radius output is designed to be similar to that of the HFA (see Fig. 2). Once sensitivities are estimated using the RATA-Standard algorithm, calculations of other statistics on the HFA printout such as total deviation (TD), pattern deviation, mean deviation (MD), pattern standard deviation, visual field index, and probability maps are identical to the HFA, except that the Radius uses its own proprietary normative database. Notably, the Radius printout does not provide any diagnosis or suggestions on how to stage glaucoma that could have been used by graders.

Despite the Radius printout emulating the HFA printout, we only provided graders with the first row of the HFA and Radius outputs—estimated sensitivities at each test location and their corresponding cross-hatching patterns. We decided not to present TD, pattern deviation, MD, pattern standard deviation, visual field index, or probability maps to the graders because the 15 dB lower measurement limit of the Radius alters the distributions of these statistics, and neither grader was familiar with the Radius distributions. Instead, we analyzed concordance in MD separately, using Deming regression and the Pearson correlation coefficient, as MD is one of the most commonly used summary statistics for glaucoma staging (e.g., using the Hodapp-Parish-Anderson system) and in trend-based analysis.

Concordance in glaucoma staging was measured using the weighted kappa statistic. Weighted kappa is applicable in this case because “mild”, “moderate” and “severe” are ordered categories, and the difference between “mild” and “severe” is greater than the difference between “mild” and “moderate” or between “moderate” and “severe”. We did allow a fourth category of “uninterpretable” if the grader could not decide which severity category was most appropriate—the number of “uninterpretable” results was tallied but otherwise excluded from the weighted kappa analysis.

## Results

A total of 100 study participants satisfied our inclusion/exclusion criteria after consecutive enrollment across all 5 clinics, with 62 OD and 38 OS due to the right eye being the default eye when both eyes met the inclusion criteria. Precisely 50 study participants were previously diagnosed with mild or suspect glaucoma based on their historic HFA VF using Medicare definitions for severity, and precisely 50 with moderate or severe glaucoma. The Table provides additional demographics information for our study participants.

**Table.** Demographic Data


Sample Size	100
Age	
Mean (SD)	69 (11.1)
Range	(26, 84)
Gender	
Male	43
Female	55
N/A	1
Race	
White	72
Black	14
Asian	4
Other	2
N/A	7

N/A, data not available; SD, standard deviation.

### Comparing Estimated Sensitivities

Our first analysis of the sensitivity data was to apply the Wilcoxon rank sum test with Bonferroni correction to OD versus OS VFs from SITA-Standard and RATA-Standard to determine if it was justified to combine OD and OS data for further analysis. SITA-Standard OD and OS were significantly different (smallest p-value was 0.00039, which is less than the Bonferroni corrected α = 0.000926), whereas RATA-Standard OD and OS samples were not significantly different (smallest *P* value was 0.041). Given this SITA-Standard result, we decided to analyze OD and OS distributions of estimated sensitivities separately. For OD, there was no statistically significant difference (smallest *P* value = 0.017) between SITA-Standard and RATA-Standard, whereas for OS there was a statistically significant difference (smallest *P* value = 0.00012)—the OS discrepancy is expected given that there was an OD versus OS discrepancy for SITA-Standard. There were no statistically significant differences between test and retest for both SITA-Standard and RATA-Standard, irrespective of the eye tested (all *P* values ≥ 0.19).

**Figure 3.**
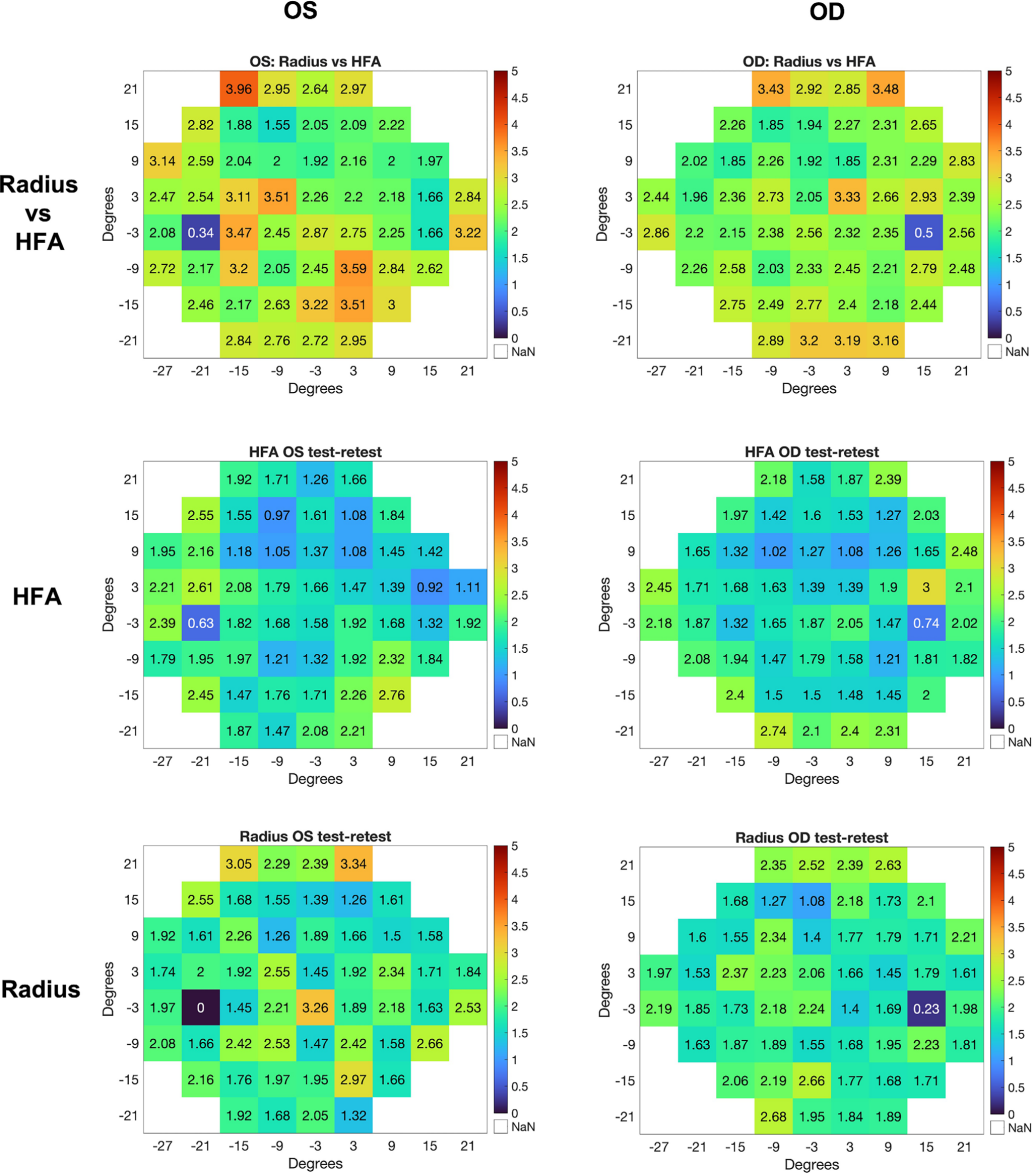
Mean absolute differences in dB for each 24-2 test location for OS (*left column*), OD (*right column*), Radius versus HFA (*top row*), HFA test versus retest (*middle row*), and Radius test versus retest (*bottom row*).

Figure 3 shows the mean absolute differences in dB at each test location of the 24-2 test pattern for OS (left column) and OD (right column), RATA-Standard versus SITA-Standard (top row), SITA-Standard test versus retest (middle row), and RATA-Standard test versus retest (bottom row). The overall mean absolute differences for OD and OS across all 54 test locations were: 2.46 dB and 2.53 dB between SITA-Standard and RATA-Standard; 1.77 dB and 1.71 dB for SITA-Standard test-retest; and 1.88 dB and 1.96 dB for RATA-Standard test-retest. The Wilcoxon rank sum test with Bonferroni correction (with α = 0.000926) showed no significant differences in absolute differences across 54 test locations between OS and OD for RATA-Standard versus SITA-Standard (*P* = 0.52), test-retest for SITA-Standard (*P* = 0.59), and test-retest for RATA-Standard (*P* = 0.70). The absolute differences between RATA-Standard and SITA-Standard were significantly different from test-retest for both SITA-Standard (*P* < 10^−10^ for both OS and OD) and RATA-Standard (*P* < 10^−6^ for OS and *P* < 10^−9^ for OD). This is expected: RATA-Standard and SITA-Standard disagree more with each other than with themselves.

The primary value of Figure 3 is not in assessing statistical significance of differences between RATA-Standard and SITA-Standard — it is better to perform hypothesis testing on the actual estimated sensitivities rather than on absolute differences. Instead, Figure 3 is more useful for assessing the clinical relevance of expected differences in dB when using RATA-Standard versus SITA-Standard. Although difference of ∼2.5 dB between SITA-Standard and RATA-Standard sensitivities may be clinically relevant in certain situations, this difference is not much greater than the ∼1.75 dB difference between SITA-Standard test and retest.

**Figure 4.**
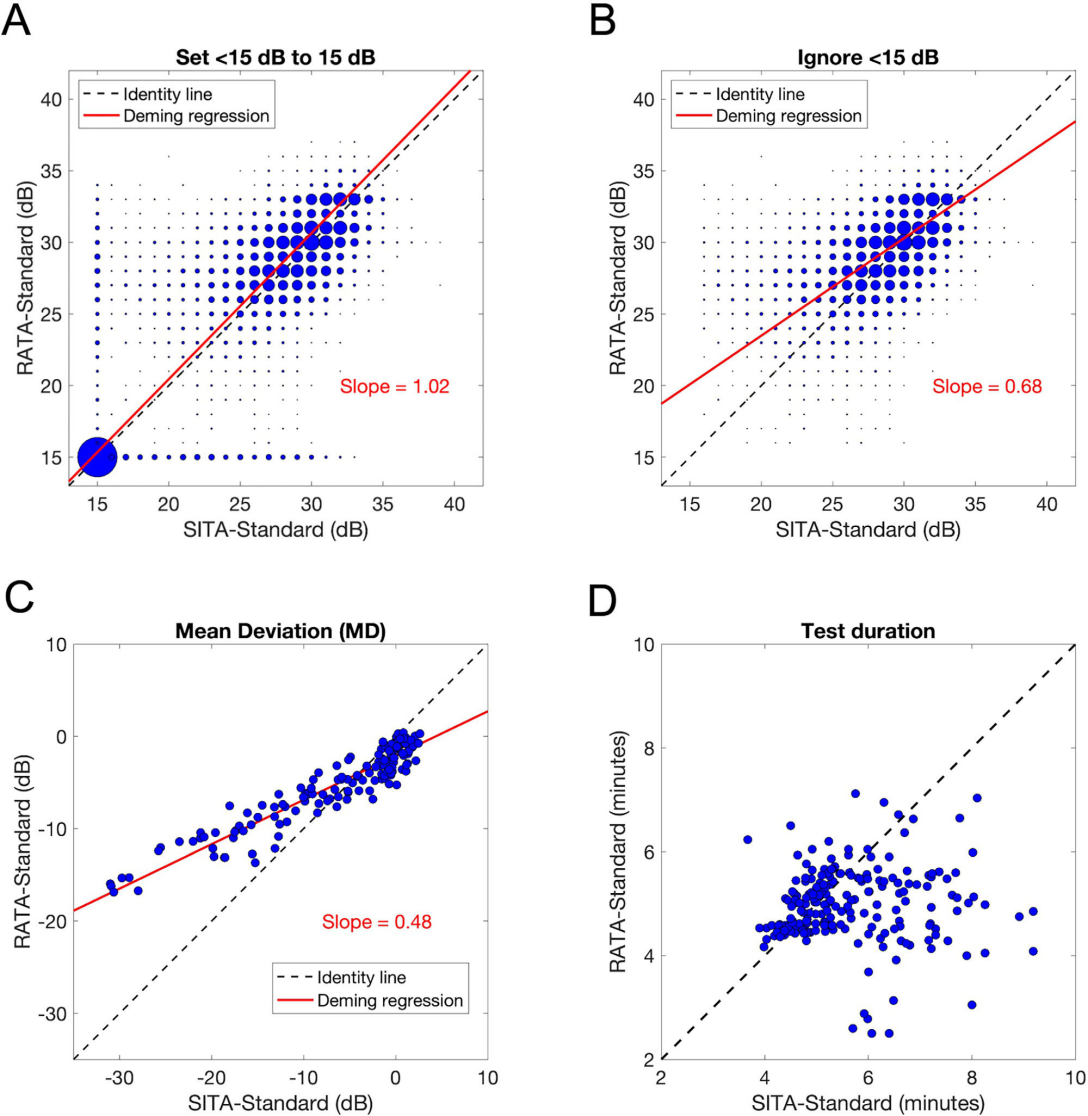
(**A**) Deming regression (*red line*) applied to all sensitivity data combined, with <15 dB set to 15 dB. The area of each data point (*blue circles*) is proportional to the number of occurrences; (**B**) Deming regression when ignoring all <15 dB data; (**C**) Deming regression on MD data; (**D**) Comparison of test duration.

Figure 4A shows the result of applying Deming regression (red line) to estimated sensitivities across all test locations when <15 dB is set to 15 dB (there were no >40 dB values). The area of the individual data points (blue circles) is proportional to the number of occurrences. The slope was 1.02 with an intercept of 0.05 dB. Figure 4B shows the analogous result when removing all <15 dB from analysis. The slope is shallower at 0.68. However, restricting the analysis to sensitivities >22 dB leads to a Deming regression slope of 0.99. In other words, the primary difference in estimated sensitivities across all test locations between RATA-Standard and SITA-Standard is that RATA-Standard estimates higher sensitivities in the 15 to 22 dB range compared to SITA-Standard. We offer a possible explanation for this phenomenon in the Discussion section.

Figure 4C compares MD between the RATA-Standard and SITA-Standard. The Pearson correlation coefficient is 0.94, whereas the slope of the Deming regression line is 0.48 with an intercept of –2.08 dB. The slope of 0.48 justifies our decision not to present TD or MD to graders for staging, as the clinician graders in this study were intimately familiar with SITA-Standard TD and MD and not RATA-Standard. There are likely two reasons for the shallower slope: (1) Figure 4B suggests that RATA-Standard produces smaller absolute TD values than SITA-Standard when the estimated sensitivity is above the 15 dB limit, and (2) lower MD values are often associated with larger scotomas—the Radius cannot detect a change if the estimated sensitivity decreases from <15 dB to an even lower value, but it can detect the change if scotoma size increases, albeit using fewer TD values.

Figure 4D shows that RATA-Standard was on average faster than SITA-Standard: 298 seconds compared to 341 seconds. Test durations were similar when SITA-Standard took four to six minutes (typical for normal VFs), but RATA-Standard was faster when SITA-Standard took more than six minutes (typical for larger scotomas). This result is expected because it takes fewer trials in a staircase procedure to reach a 15 dB measurement floor than a 0 dB measurement floor, which means that the Radius is expected to take less time to detect large scotomas.

### Concordance in Glaucoma Staging

**Figure 5.**
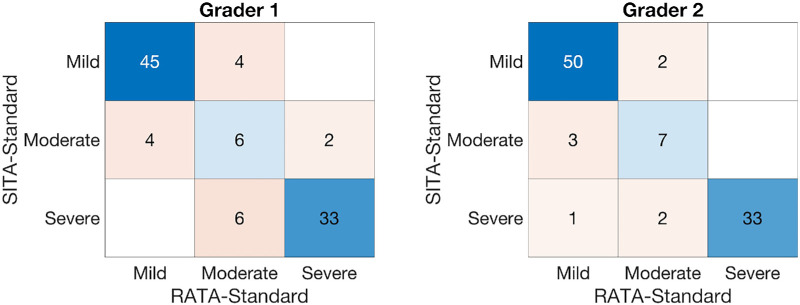
Confusion matrices for grader 1 and grader 2.

Our second primary outcome measure was concordance in glaucoma staging between SITA-Standard and RATA-Standard given the estimated sensitivities. For grader 1, κ = 0.91 while it was κ = 0.93 for grader 2. Generally, κ ≥ 0.81 is considered to be “near perfect agreement”. Figure 5 shows the confusion matrices for the two graders. Note that grader 2 had a total of 98 comparisons, not 100, because grader 2 labeled two SITA-Standard test-retest results as “uninterpretable”; there were no “uninterpretable” for RATA-Standard. Also note that while we had precisely 50 study participants who were diagnosed as mild or suspect glaucoma based on a historic SITA-Standard VF (inclusion criteria), staging based on test-retest SITA-Standard VFs showed 49 mild for grader 1 and 52 mild for grader 2. Finally, agreement between graders was also high: κ = 0.93 for SITA-Standard and κ = 0.92 for RATA-Standard.

### Reliability Indexes

The Wilcoxon rank sum test showed statistically significant differences between distributions of all three reliability indices. RATA-Standard had a significantly lower FP% (*P* = 0.00078) with a mean of 1.62% compared to 3.65% for SITA-Standard. RATA-Standard also had a significantly lower FL% (*P* = 0.03) with a mean of 9.7% compared to 11.5% for SITA-Standard. However, FN% for RATA-Standard was significantly higher (*P* = 1.2 × 10^−^^6^) with a mean of 9.3% compared to 3.5% for SITA-Standard. At least one study has suggested that FP% is the most important reliability index for glaucoma.^13^ However, we note that comparisons of FP%, FL% and FN% between SITA-Standard and RATA-Standard are problematic given that precise details of how SITA-Standard measures these reliability indexes are not published.^14^

## Discussion

This study demonstrates that RATA-Standard is comparable to SITA-Standard in several important ways: (1) estimated sensitivities at individual test locations were not significantly different for OD, (2) estimated sensitivities for OS were significantly different, however this was likely due to SITA-Standard OD and OS being significantly different—most likely an artifact of our sample and not to be expected in general, (3) the slope of the Deming regression line was very close to 1 when interpreting <15 dB as 15 dB, and (4) concordance in glaucoma staging was high, with κ ≥ 0.91 for both graders. There were also several areas where RATA-Standard outperformed SITA-Standard in our sample: no significant difference between OS and OD, faster test times, and no “uninterpretable” VFs when staging glaucoma.

It is important to understand why we interpret the statistically significant difference between SITA-Standard OD and OS to be an artifact of our sample. First, statistical significance was due to only one test location in the periphery (at [−9, −21] of the 24-2 test pattern for OD, or [9, −21] for OS) and not a cluster of points, which would suggest a more systematic difference. Second, the difference was observed between eyes from different patients, not eyes from the same patient. If the observed SITA-Standard OD versus OS difference is real, then the implication is that glaucoma—the disease itself—progresses differently in left and right eyes of different patients at precisely one test location in the 24-2 test pattern. Such a claim requires extraordinary evidence from a very large sample of glaucomatous eyes, which is why we attribute the significant difference between OD and OS for SITA-Standard in our sample to an artifact that is unlikely to be replicated with a larger sample of eyes.

The correlation between RATA-Standard MD values and SITA-Standard MD values was also high at 0.94, suggesting that RATA-Standard MD can be mapped to SITA-Standard MD, as well as vice versa, using the equation of the Deming regression line in Figure 4C. For example, the Hodapp-Parish-Anderson system for staging glaucoma severity can be modified for the Radius by setting MD > −5 dB for mild, −5 dB ≥ MD ≥ −8 dB for moderate, and −8 dB > MD for severe — the Deming regression line in Figure 4C maps −6 dB on the HFA to −4.96 dB on the Radius, and −12 dB on the HFA to −7.84 dB on the Radius. The high correlation in MD also suggests that trend-analysis using MD may work relatively well despite the 15 dB lower measurement limit. However, the distribution of rates of MD change will likely be different, and previously published values for seventy-fifth percentile (moderate) and ninetieth percentile (rapid) MD worsening for SITA-Standard must be recomputed for RATA-Standard.

One interesting observation is that the slope of the Deming regression line for pointwise estimated sensitivities across all test locations values was 0.68 when ignoring all <15 dB (Fig. 4B) but was 0.99 when ignoring all <23 dB. One possible explanation lies in how sensitivities are estimated mathematically. Typically in psychophysics, stimuli with intensity levels both above and below the actual sensitivity value are presented, which helps the algorithm (e.g., a staircase procedure) hone in on the sensitivity value. Close to the measurement floor, this becomes difficult to do, increasing the uncertainty (and the variance) in the estimate. Higher variance in estimated sensitivities near the 15 dB measurement floor of the Radius results in either estimating <15 dB or estimating a higher dB value than SITA-Standard. This helps explain the 0.68 slope, which ignores all <15 dB.

Important innovations in this study compared to previous VRP validation studies are (1) hypothesis testing of differences between distributions of estimated sensitivities across all test locations and (2) a clinical component where we measured concordance in glaucoma staging for masked graders, who are seasoned glaucoma specialists. Regarding testing differences in estimated sensitivities at all test locations, a more detailed level of analysis is essential for validation of wearable VRPs because they are currently limited in power compared to the HFA—unless HFA test parameters are modified, reduced power implies reduced measurement range. Many commercially available wearable VRPs lower background luminance into the mesopic range to increase measurement range. However, both rods and cones are active in the mesopic range, unlike testing in the photopic range where only cones are active, and there is no a priori reason for estimated sensitivities of such systems to be similar to the HFA, even if summary statistics such as MD might be comparable.

Regarding using a common clinical endpoint such as Medicare approved severity classification based on assessments by individual masked clinician graders, as stated earlier: concordance in summary statistics does not imply concordance in estimated sensitivities at individual test locations. In other words, summary statistical analysis does not take into account classic patterns of glaucoma which clinicians can instinctively identify when analyzing a test. This component is critically important when managing a patient at risk for or with manifest glaucoma field damage.

Major strengths of this study include not just the more detailed statistical analysis, but also the large sample size from five different clinics: 100 eyes × 2 VFs × 54 test locations = a sample size of 10,800 estimated sensitivities for both the Radius and the HFA. Major limitations of this study are primarily hardware related: current power limitations result in a 15 dB lower measurement limit for the Radius headset. Because of this limitation, we set all <15 dB to 15 dB to avoid excluding data for analysis. While setting <15 dB to 15 dB may conceal differences that would be observed if sensitivities <15 dB were estimated by the Radius, it is important to note that this issue with data analysis would exist even if two sets of HFA test results were compared: <0 dB is a range, not a sensitivity, and no commonly used hypothesis test applies to both discrete values and ranges. The alternative would have been to ignore all cases where either the Radius or the HFA estimated <15 dB or <0 dB. However, this would have removed clinically important data (scotomas) from the analysis.

Another issue with having a 15 dB lower limit is that the Radius may not be able to accurately characterize severe or worsening VF defects for sensitivities <15 dB, which may limit its application for following progression in some cases of severe glaucoma. The HFA has this problem for <0 dB. Figure 4C shows that in the majority of cases, worsening below 15 dB at a given test location is accompanied by an enlargement of the scotoma that the Radius can detect using MD. This suggests that the Radius may be useful for monitoring eyes with relatively severe disease, and not just ocular hypertensives or glaucoma suspects. However, robust precaution is necessary when the Radius estimates sensitivities <15 dB, whether for single field analysis or longitudinal follow-up. There are also rare cases where sensitivity progressively decreases below 15 dB at a given test location without affecting sensitivities at neighboring test locations. We note that future hardware developments should be able to address both the 15 dB and 40 dB limitations.

Other limitations of this study include not presenting the entire printed outputs of the HFA and Radius for staging, and not demonstrating concordance in detecting glaucoma worsening. The result in Figure 4C is encouraging for trend-based analysis, but it is unclear what the effect the 15 dB measurement limit has on event-based analysis. The sample of patients in this study may not be representative of glaucoma in the general population given our inclusion/exclusion criteria, which could potentially limit clinical applicability. For example, we preselected patients with reliable VFs. The resulting ethnic breakdown of our test subjects may also differ from the patient population at different clinics. Future studies will be required to address these issues. This is also true for the interesting observation that one grader labeled one pair of SITA-Standard VFs as “uninterpretable” although there were no “uninterpretable” VFs for RATA-Standard.

Wearable VRPs have the potential to become the new paradigm in VF testing by improving patient experience, clinical workflow, and in general enabling VFs to be measured more frequently, which improves the accuracy of diagnosis. Recent developments with VF testing in a metaverse environment^15^ also suggest that VRP may play a crucial role in telehealth—monitoring disease progression without an in-person visit. To realize this potential, it is necessary to demonstrate statistical noninferiority to a clinical standard such as the HFA. This study demonstrates that the RATA-Standard algorithm is comparable to the SITA-Standard algorithm, both in terms of estimated sensitivities between 23–40 dB, and in terms of glaucoma staging.
